# The Price of Radium

**Published:** 1923-12

**Authors:** 

**Affiliations:** 43, Parker Street, Kingsway, W.C. 2


### The Price of Radium.

SIR,—In your November issue you state that the " Belgian Company " offers radium at about £4 a milligram.

As representatives in the British Empire of the Radium Belge (Union Minière du Haut Katanga), we would like to have an opportunity of correcting this statement. Previous to the discovery of the rich ores in the Belgian Congo, the standard price for radium element was $110 per milligram, and this price was reduced following the production of radium from the Congo ore to $70, equivalent at the rate of exchange at the time of writing—4.37 dollars to the pound sterling—to £16 0s. 4d.

It is not anticipated that radium will be available at a lower price than this.

WATSON AND SONS (ELECTRO-MEDICAL), LTD.

43, Parker Street,
Kingsway, W.C. 2.

### A GREAT SYDNEY HOSPITAL.

Here we have a great hospital of 518 beds doing all the work of a London general hospital with a medical school, towards whose annual income of £100,000 in round figures subscriptions and donations supply upwards of £12,000, and the voluntary contributions of patients a like sum, notwithstanding that the institution is in receipt of a Government subsidy of more than £50,000 a year. Nor is this all. In no hospital can a finer practical example be found of the vitality of the voluntary spirit than the Royal Alfred Hospital furnishes in what is called " The Auxiliary." In this the ladies of Sydney are in the van. The auxiliary provided the hospital with the whole of the bedding and linen required for the year, valued at £3,040 ; conducted the tea room at the hospital, realising a net profit of £780 ; ran a hospital shop, where patients, visitors, nurses and other members of the staff may obtain articles which would otherwise have to be purchased outside —profit, for only seven months, £200 ; catered for the refreshments at the Royal Agricultural Show, profit £540 ; carried out systematic instruction, on the kindergarten principle, of the patients in the children's wards, and organised entertainments realising a net profit of some £2,500 !

